# Probiotics for functional constipation in children: an overview of overlapping systematic reviews

**DOI:** 10.3389/fcimb.2023.1323521

**Published:** 2024-01-08

**Authors:** Yunxin Zhang, Aiping Li, Jing Qiu, Hua Wen, Hanwen Zhang, Xiangjuan Sun

**Affiliations:** ^1^ Hospital of Chengdu University of Traditional Chinese Medicine, Chengdu, China; ^2^ Guang’an Hospital of Traditional Chinese Medicine, Guangan, China; ^3^ Chengdu University of Traditional Chinese Medicine, Chengdu, China

**Keywords:** children, functional constipation, probiotics, evidence, overview

## Abstract

**Background:**

This overview of systematic reviews (SRs) and meta-analysis (MAs) aimed to systematically collate, appraise and synthesize evidence of probiotics for functional constipation (FC) in children.

**Methods:**

SRs/MAs of probiotics for FC in children were systematic identified by searching Cochrane Library, PubMed, Embase, and Web of science. Assessment of Multiple Systematic Reviews 2 (AMSTAR-2), Preferred Reporting Items for Systematic Reviews and Meta-Analyses (PRISMA), and Grading of Recommendations, Assessment, Development, and Evaluation (GRADE) were unitized by two reviewers independently to assess the methodological quality, reporting quality, and quality of evidence, respectively.

**Results:**

Seven SRs/MAs met the eligibility criteria and were included in this study. According to AMSTAR-2, a very low methodological quality assessment was given to the included SRs/MAs due to the limitations of items 2, 4 and 7. For the PRISMA statement, the overall quality of reporting was unsatisfactory due to the lack of reporting on protocol, risk of bias across studies, synthesis of results, and additional analysis. According to GRADE, the quality of evidence for outcomes was rated as very low to moderate.

**Conclusions:**

Probiotics may be beneficial in improving FC in children. Because of limitations and inconsistent conclusions, further rigorous, normative and comprehensive SRs/MAs are needed to provide robust evidence for definitive conclusions.

## 1 Introduction

Functional constipation (FC) is a highly prevalent disorder in children with a cumulative global prevalence of 9.5% (Koppen I. et al., 2018). FC has been reported to be responsible for 25% of pediatric gastroenterologist visits and 3% of general pediatric visits (Banaszkewicz and Szajewska, 2005). Suffering from FC not only reduces the quality of life of children, but also places a heavy burden on children and their families, resulting in increased medical costs (Liem et al., 2009; Vriesman et al., 2019). In addition, about 25% of children with FC continue to experience symptoms as adults (Bongers et al., 2010). Currently, laxatives such as polyethylene glycol are the first-line drugs currently used in the clinical management of FC in children (Tabbers et al., 2014). However, due to the discomfort, ineffectiveness, and emotional toll of the disease, children frequently do not adhere to laxative therapy in an acceptable manner (Koppen IJN. et al., 2018). As a result, approximately 40% of children with FC presenting to gastroenterology departments use complementary medicines (Vlieger et al., 2008; Jandhyala et al., 2015).

The definition of probiotics is “living microorganisms that confer health benefits to the host when administered in sufficient amounts” (Hill et al., 2014). Probiotics that are available include, but are not limited to, *yeasts, Lactobacillus casei, Lactobacillus reuteri, Bifidobacterium infantis, Bifidobacterium longum, Bifidobacterium rhamnosus G.G., Bacillus clausii, Bacillus coagulans, Lactobacillus paracasei*, and *Bifidobacterium infantis* (Jain et al., 2023). It is now recognized that an ideal probiotic must possess the following properties: (a) it must exist as live cells, ideally in huge amounts, and must be safe, non-pathogenic, non-toxic, and non-invasive; (b) it should be a strain that can benefit the host by promoting faster growth or resistance to illness; (c) it should be hardy and able to survive in the intestinal environment; and (d) it must be stable and able to survive for an extended period of time, either in storage or on the field (Fuller and Tannock, 1999).

Taking probiotic supplements can alter the intestinal microbiota, influence intestinal peristalsis, and regulate the intestinal environment, thus reducing the symptoms of FC (Dimidi et al., 2017). It has been proposed that the intake of probiotics should be considered as one of the key strategies for the restoration of intestinal dysfunction in children (Savino et al., 2017). The number of systematic reviews (SRs) and meta-analyses (MAs) evaluating probiotics for the treatment of FC in children is increasing. However, the results of these overlapping studies have been inconsistent and of varying quality, which poses a challenge to the use of probiotics as adjunctive therapy for FC in children. To comprehensively collect, evaluate and synthesize the evidence from SRs/MAs in this field, we carried out this overview.

## 2 Methods

### 2.1 Criteria for inclusion and exclusion

Studies were screened for inclusion using the following criteria: (a) SR/MAs based on randomized controlled trials (RCTs); (b) individuals who met the Rome III/IV criteria for FC and were under the age of 18; (c) the intervention group consisted of probiotics taken alone or in combination with laxatives, whereas the control group consisted of placebo or laxatives; (d) outcomes included treatment success, stool consistency, stool frequency, abdominal pain, and adverse events.

### 2.2 Strategy for searching

A comprehensive search for SRs/MAs of probiotics for FC in children from database creation to October 2023 was carried out in Cochrane Library, Web of Science, Embase, and PubMed. Children, functional constipation, probiotics, and systematic review were applied as search terms.

### 2.3 Data collection and extraction

The literature obtained from the search was independently screened by two reviewers using a tier-by-tier screening principle to obtain literature that ultimately met the inclusion criteria. First, all retrieved documents were imported into the EndNote 8 software, and then duplicates were automatically identified and removed by the software. Subsequently, we read the titles and abstracts of the remaining documents to further exclude literature that did not meet the inclusion criteria. Finally, we read the full text of the remaining documents to determine whether they met the final inclusion criteria.

The first author, publication year, nation, sample size, trial quality, interventions, methods of quality evaluation, and a summary of the intervention effects were retrieved from the included reviews.

### 2.4 Methodological quality assessment

Through the use of Assessment of Multiple Systematic Reviews-2 (AMSTAR-2) by two reviewers independently, the methodological quality of the included reviews was assessed (Shea et al., 2017). AMSTAR-2 contains 16 items, and 7 of which are key items. Each item can be rated on three levels, which are “yes”, “partially yes”, and “no”. When more than one critical component is not met, methodological quality is extremely poor, low when only one critical component is not met, moderate when more than one non-critical component is not met, and high when neither no or just one non-critical component is not fulfilled (Shea et al., 2017).

### 2.5 Reporting quality assessment

Through the use of Preferred Reporting Items for Systematic Reviews and Meta-Analyses (PRISMA) by two reviewers independently, the reporting quality of the included reviews was assessed (Page et al., 2021). The PRISMA consists of 27 items, each of which is rated as “no” (not reported), “yes” (fully reported), or “partially yes” (partially reported) (Page et al., 2021).

### 2.6 Evidence quality assessment

Through the use of Grades of Recommendation, Assessment, Development and Evaluation (GRADE) by two reviewers independently, the evidence quality of the included reviews was assessed (Pollock et al., 2016). Evidence could be downgraded owing to publication bias, inconsistency, indirectness, imprecision, or risk of bias. The level of evidence quality can be classified as “high,” “very low,” “low,” or “moderate.” (Pollock et al., 2016).

## 3 Results

### 3.1 Selection of literature

**Figure 1:**
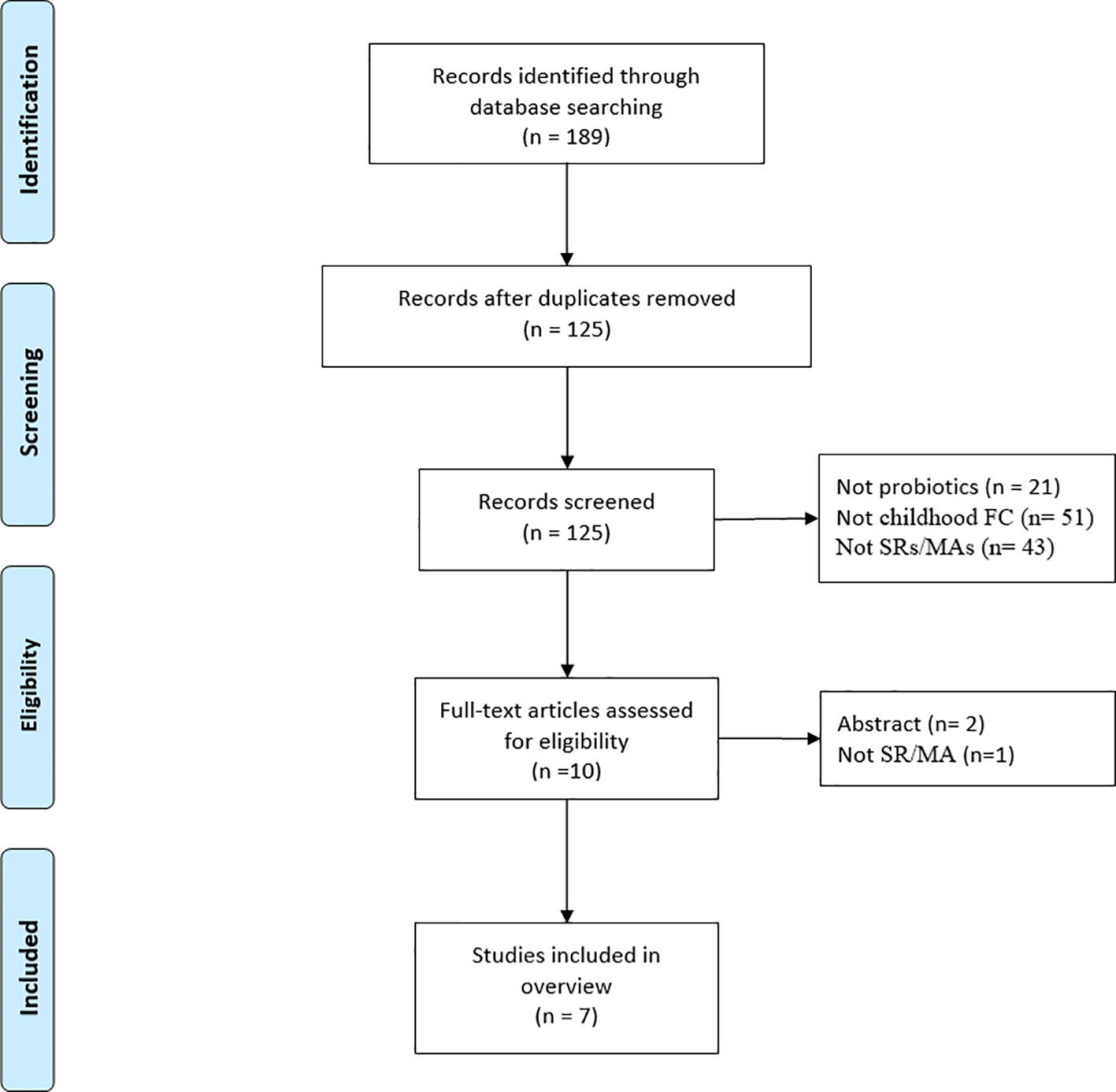
Publication selection procedure.

A total of 189 articles were initially obtained from the database (**Figure 1**), leaving 125 articles after removing duplicates. Then, 115 articles were disqualified after their titles and abstracts were scrutinized, and three articles were disqualified after their full texts were examined. Seven SRs/MAs (Chmielewska and Szajewska, 2010; Huang and Hu, 2017; Jin et al., 2018; Gomes and Morais, 2019; Wu, 2020; Wallace et al., 2022; Liu et al., 2023) ultimately met the requirements for inclusion in this review.

### 3.2 Study characteristics

As shown in **Table 1**, the included studies were published between 2010 and 2023. Of the seven included studies, four were conducted in China and the other three in Poland, the Brazil, and England. Sample sizes for SRs/MAs varied widely (111 to 1504). The control group was treated by probiotics or probiotics plus laxatives, and placebo or laxatives was used in the control group. Summary of intervention of the included reviews is given in **Table 2**.

**Table 1:** Characteristics of the included reviews.

Studies	Country	Trials (subjects)	Diagnostic criteria	Experimental Intervention	Control Intervention	Outcomes	Conclusion summary
Chmielewska and Szajewska, 2010	Poland	2 (111)	Rome III	Probiotics	Placebo	① ,③,④	Until more data are available, we believe the use of probiotics for the treatment of FC in children should be considered investigational.
Huang and Hu, 2017	China	6 (444)	Rome III	Probiotics	Placebo	①,②	Probiotics increase stool frequency and have beneficial effects in Asian children. However, caution is needed when interpreting these outcomes because of the existence of heterogeneity.
Jin et al., 2018	China	4 (382)	Rome III	Probiotics	Placebo	③	The use of probiotics was associated with significant improvement in glycerin enema use and abdominal pain but did not affect the treatment success and other function indices.
Gomes and Morais, 2019	Brazil	8 (564)	Rome III	Probiotics	Placebo	① ,②	Despite the probiotics’ positive effects on certain characteristics of the intestinal habitat, there is still no evidence to recommend it in the treatment of constipation in children.
Wu, 2020	China	3 (641)	Rome III	Probiotics	Placebo	①,②	There were significant differences in bowel movements between the experimental and placebo groups, and no significant differences in stool consistency. In addition, there were no adverse effects of the *L. reuteri* DSM 17938 treatment reported.
Wallace et al., 2022	England	14 (1127)	Rome III, Rome IV	Probiotics, probiotics+ laxatives	Placebo, laxatives	①,③,④	There is insufficient evidence to conclude whether probiotics are efficacious in successfully treating chronic constipation without a physical explanation in children or changing the frequency of defecation.
Liu et al., 2023	China	17 (1504)	Rome III, Rome IV	Probiotics, probiotics+ laxatives	Placebo, laxatives	①,②,③,⑤	Current evidence did not advocate using probiotics in treating FC in children. Additional rigorous evidence is required to evaluate and establish the effectiveness and safety of probiotics for childhood FC.

① : Stool frequency; ②: stool consistency; ③: treatment success; ④: adverse events; ⑤: abdominal pain.

**Table 2:** Summary of intervention of the included reviews.

Studies	Probiotic		Comparison		Duration
	Type	Dose	Type	Dose	
Chmielewska and Szajewska, 2010	*E. coli* Nissle 1917, *L. casei* Shirota*, L. rhamnosus* Lcr35, *B. lactis* DN-173 010, *Lactobacillus rhamnosus* GG, *Lactobacillus* Sporogenes, *B. breve, L.acidophilus, B. infantis*	2-25×10^9^ CFU, 8×10^8^ CFU	Placebo	Not applicable	Once or twice daily, 2-12 weeks,
Huang and Hu, 2017	*L. rhamnosus* Lcr35, *B. lactis* DN-173 010, *Lactobacillus casei, Lactobacillus rhamnosus*, *Lactobacillus rhamnosus* GG	1×10^9^ CFU, 1.2-8×10^8^ CFU	Placebo	Not applicable	Once or twice daily, 3-12 weeks,
Jin et al., 2018	*L. rhamnosus* Lcr35, *B. lactis* DN-173 010	4.25×10^9^ CFU, 8×10^8^ CFU	Placebo	Not applicable	Once or twice daily, 3-12 weeks,
Gomes and Morais, 2019	*L. reuteri* DSM17938, *B. longum*, *B. lactis* DN-173 010, *L. rhamnosus* Lcr35, *L. rhamnosus* GG ATCC 531032	1-4.25×10^9^ CFU, 1-8×10^8^ CFU	Placebo	Not applicable	Once or twice daily, 3-12 weeks,
Wu, 2020	*L. reuteri* DSM17938	1-7×10^8^ CFU	Placebo	Not applicable	Once or twice daily, 4-15 weeks,
Wallace et al., 2022	*bacillusrhamnosus* GG, *Lactobacillus casei, L rhamnosus, L plantarum, Bifidobacterium lactis*, *L. rhamnosus* Lcr35, *Clostridium butyricum Miyairi, L. reuteri* DSM17938	1-4×10^9^ CFU, 1-8×10^8^ CFU	Laxatives; MgO; macrogol	1-3ml/kg/day; 50 mg/kg/day; 10 g/day	Once or twice daily, 4-12 weeks
Liu et al., 2023	*L. reuteri* DSM17938, *B. longum*, *B. lactis* DN-173 010, *L. rhamnosus* Lcr35, *L. acidophilus* DDS-1, *S. boulardii*, *Lactobacillus reuteri, Lactobacillus rhamnosus*	1-5×10^9^ CFU, 1-8×10^8^ CFU	Laxatives; MgO; macrogol	1-3ml/kg/day; 50 mg/kg/day; 10 g/day	Once or twice daily, 3-12 weeks,

### 3.3 Results of quality assessment

#### 3.3.1 Results of methodological quality

The AMSTAR-2 assessment of the methodological quality is shown in **Table 3**. Deficiencies that undermine the quality of the methodology included items 2 (lack of the protocol being registered), 4 (inadequate search strategies for each database), and 7 (absence of a list of trials that were excluded). Notably, six of the seven included studies had one or more key items that did not meet the specifications and were therefore rated as low or very low methodological quality, and their results overall dominated the methodological quality of probiotics for FC in children. More detailed information is given in **Table 3**.

**Table 3:** Quality assessment of the included reviews by the AMSTAR-2 tool.

Author, Year	AMSTAR-2																Quality
	Q1	Q2	Q3	Q4	Q5	Q6	Q7	Q8	Q9	Q10	Q11	Q12	Q13	Q14	Q15	Q16	
Chmielewska and Szajewska, 2010	Y	N	Y	Y	Y	Y	Y	Y	Y	Y	Y	Y	Y	Y	Y	Y	Low
Huang and Hu, 2017	Y	N	Y	Y	Y	Y	N	Y	Y	Y	Y	Y	Y	Y	Y	Y	Very low
Jin et al., 2018	Y	N	Y	Y	Y	Y	N	Y	Y	Y	Y	Y	Y	Y	Y	Y	Very low
Gomes and Morais, 2019	Y	N	Y	Y	Y	Y	N	Y	Y	Y	Y	Y	Y	Y	Y	Y	Very low
Wu, 2020	Y	N	Y	PY	Y	Y	N	Y	Y	Y	Y	Y	Y	Y	Y	Y	Very low
Wallace et al., 2022	Y	Y	Y	Y	Y	Y	Y	Y	Y	Y	Y	Y	Y	Y	Y	Y	High
Liu et al., 2023	Y	Y	Y	Y	Y	Y	N	Y	Y	Y	Y	Y	Y	Y	Y	Y	Low

Y, yes; PY, partial yes; N, no.

#### 3.3.2 Results of reporting quality

Using the PRISMA checklist, 28.57% of reviews lacked detailed information on Q5 (protocol and registration), 71.43% on Q14 and Q21 (synthesis of results), 71.43% on Q15 and Q22 (risk of bias across studies), and 71.43% on Q16 and Q23 (additional analyses). All other items were completely reported. More detailed information is given in **Table 4**.

**Table 4:** Results of the reporting quality.

Section/topic	Items	Chmielewska, 2010	Huang, 2017	Jin, 2018	Gomes and Morais, 2019	Wu, 2020	Wallace, 2022	Liu, 2023	Compliance (%)
Title	Q1. Title	Y	Y	Y	Y	Y	Y	Y	100%
Abstract	Q2. Structured summary	Y	Y	Y	Y	Y	Y	Y	100%
Introduction	Q3. Rationale	Y	Y	Y	Y	Y	Y	Y	100%
	Q4. Objectives	Y	Y	Y	Y	Y	Y	Y	100%
Methods	Q5. Protocol and registration	N	N	N	N	N	Y	Y	28.57%
	Q6. Eligibility criteria	Y	Y	Y	Y	Y	Y	Y	100%
	Q7. Information sources	Y	Y	Y	Y	Y	Y	Y	100%
	Q8. Search	Y	Y	Y	Y	Y	Y	Y	100%
	Q9. Study selection	Y	Y	Y	Y	Y	Y	Y	100%
	Q10. Data collection process	Y	Y	Y	Y	Y	Y	Y	100%
	Q11. Data items	Y	Y	Y	Y	Y	Y	Y	100%
	Q12. Risk of bias in individual studies	Y	Y	Y	Y	Y	Y	Y	100%
	Q13. Summary measures	Y	Y	Y	Y	Y	Y	Y	100%
	Q14. Synthesis of results	N	Y	Y	Y	N	Y	Y	71.43%
	Q15. Risk of bias across studies	N	Y	Y	Y	N	Y	Y	71.43%
	Q16. Additional analyses	N	Y	Y	Y	N	Y	Y	71.43%
Results	Q17. Study selection	Y	Y	Y	Y	Y	Y	Y	100%
	Q18. Study characteristics	Y	Y	Y	Y	Y	Y	Y	100%
	Q19. Risk of bias within studies	Y	Y	Y	Y	Y	Y	Y	100%
	Q20. Results of individual studies	Y	Y	Y	Y	Y	Y	Y	100%
	Q21. Synthesis of results	N	Y	Y	Y	N	Y	Y	71.43%
	Q22. Risk of bias across studies	N	Y	Y	Y	N	Y	Y	71.43%
	Q23. Additional analysis	N	Y	Y	Y	N	Y	Y	71.43%
Discussion	Q24. Summary of evidence	Y	Y	Y	Y	Y	Y	Y	100%
	Q25. Limitations	Y	Y	Y	Y	Y	Y	Y	100%
	Q26. Conclusions	Y	Y	Y	Y	Y	Y	Y	100%
Funding	Q27. Funding	Y	Y	Y	Y	Y	Y	Y	100%

Y, yes; PY, partial yes; N, no.

#### 3.3.3 Results of evidence quality

Evidence quality of the 11 outcome indicators from included reviews was summarized by the GRADE system (**Table 5**). The overall level of evidence quality ranged from moderate to low. Inconsistency was the first element that led to downgrading the evidence quality, followed by risk of bias and imprecision. More detailed information is presented in **Table 5**.

**Table 5:** Results of evidence quality.

Review	Outcomes	№ of trails	Certainty assessment					№ of patients		Relative effect (95% CI)	Quality
			Limitations	Inconsistency	Indirectness	Imprecision	Publication bias	Experimental	Control		
Huang and Hu, 2017	Stool frequency	6	Serious^a^	Serious^b^	No	No	No	231	213	MD 0.73 [0.14, 1.31]	⨁⨁⨁◯◯ Low
	Stool consistency	3	No	Serious^b^	No	Serious^c^	No	133	134	MD -0.07 [-0.21, 0.06]	⨁⨁⨁◯◯ Low
Jin et al., 2018	Treatment success	4	No	Serious^b^	No	Serious^c^	No	188	176	RR 1.05 [0.81, 1.38]	⨁⨁⨁◯◯ Low
Wu, 2020	Stool frequency	5	Serious^a^	No	No	No	No	373	364	MD 1.12 [0.85, 1.39]	⨁⨁⨁⨁◯ Moderate
	Stool consistency	3	No	No	No	Serious^c^	No	107	106	MD 0.98 [0.66, 1.44]	⨁⨁⨁⨁◯ Moderate
Wallace et al., 2022	Treatment success	4	Serious^a^	Serious^b^	No	No	No	161	152	RR 1.29 [0.73, 2.26]	⨁⨁⨁◯◯ Low
	Adverse events	5	Serious^a^	No	No	No	No	194	177	RR 0.64 [0.21, 1.95]	⨁⨁⨁⨁◯ Moderate
Liu et al., 2023	Stool frequency	2	No	No	No	Serious^c^	No	85	85	SMD 0.40 [0.10, 0.70]	⨁⨁⨁⨁◯ Moderate
	Stool consistency	2	Serious^a^	No	No	Serious^c^	No	113	113	OR 0.53 [0.29, 0.96]	⨁⨁⨁◯◯ Low
	Treatment success	3	Serious^a^	No	No	No	No	222	144	OR 1.54 [0.90, 2.61]	⨁⨁⨁⨁◯ Moderate
	Abdominal pain	2	No	Serious^b^	No	Serious^c^	No	113	113	OR 1.05 [0.57, 1.92]	⨁⨁⨁◯◯ Low

IBS-SSS, IBS symptom severity scale; QoL, quality of life. ^a^: the experimental design had a large bias in random, distributive findings or was blind; ^b^: the confidence interval overlaps less, the heterogeneity test P was very small, and the I^2^ was larger; ^c^: the Confidence interval was not narrow enough, or the simple size is too small.

## 4 Discussion

According to evidence-based medicine, evidence derived from SR/MA is considered to be at the highest level (Yang et al., 2022; Pan et al., 2023). However, not all medical evidence derived from SRs/MAs is convincing, as SRs/MAs may introduce various risks of bias in the production of evidence that undermine the reliability of their results (Huang et al., 2021; Liu et al., 2022). It is therefore important to systematically assess the quality of evidence before it is used (Huang et al., 2022a). Experts in evidence-based medicine have proposed the method of overview with the aim of assessing and synthesizing existing data on the same subject (Huang et al., 2022b). To thoroughly compile, evaluate, and summarize the most recent research on the use of probiotics for FC in children, we conducted this overview.

### 4.1 Summary of main results

AMSTAR-2, PRISMA, and GRADE were the primary evaluation tools used in this study. Almost all included studies demonstrated substantial inadequacies in both important and non-critical items, according to the AMSTAR-2 assessment results. It was also these deficiencies that led to all methodological quality being judged as very low. The items with obvious methodological flaws included item 2, item 4, and item 7. The reporting quality evaluated by PRISMA checklists identified underreporting in protocol and registration, risk of bias across studies, synthesis of results, as well as additional analyses. The GRADE assessment showed that all included outcome indicators were rated to be moderate to low quality of evidence. Inconsistency was the first element that led to downgrading the quality of evidence, followed by imprecision and risk of bias. The results of all included reviews showed that probiotic may be an effective and safe therapy for FC in children, but inconsistencies existed. Therefore, probiotics may be effective for FC in children based on the above findings, but this conclusion should be viewed with caution in light of the limitations of the available evidence.

### 4.2 Quality of SRs/MAs

AMSTAR-2 was officially promulgated in 2017 to assess the methodological quality of SRs/MAs and to promote their standardization to ensure the reliability of the evidence generated. Disappointingly, the methodological quality of most of the included studies was rated as low quality. For item 2, five studies did not register a protocol, raising the likelihood of considerable bias. Pre-registration protocols help to make the research process transparent, which is essential for methodological quality evaluation (Pan et al., 2023). For item 4, only three studies provided specific search strategies for each database, which may not be convincing enough for the adequacy of the search and reduce the reliability of the findings. The search strategy is a guarantee of recovering the search results, and the lack of providing a search strategy does not guarantee the scientific validity of the included and excluded studies. For item 7, only two reviews provided a list of excluded trials, which is likely to have resulted in the non-inclusion of eligible trials, thereby creating a risk of publication bias. A list of omitted papers is regarded crucial for high-quality SRs, and the absence of this process does not guarantee that some usable material was excluded, raising the possibility of bias.

Surprisingly, authors of the included studies lacked confidence in their SRs/MAs and were therefore reluctant to draw definitive conclusions on probiotics for FC in children. The majority of the evidence had an unsatisfactory level of certainty, according to GRADE. These findings are consistent with the authors’ concern that low-quality data often suggest that findings of SRs/MAs may not match actual results. Through further analysis of the results of evidence quality, we found that low-quality original RCTs were the common direct cause of low evidence. There is still much room for improvement in published RCTs of probiotics for the treatment of FC in children in terms of randomization, allocation concealment, or blinding bias. As evidence-based medicine continues to evolve, clinical decision making is increasingly dependent on high quality evidence. Because only high-quality RCTs are the gold standard for reducing the risk of bias when assessing the efficacy of therapies, well-planned and executed investigational trials are increasingly encouraged as a source of evidence generation.

### 4.3 Generalities of FC in children

The Rome Foundation first defined childhood FC in the Rome III criteria in 2006, and the Rome III diagnostic criteria for FC in children were updated by the Rome IV criteria in 2016 (Drossman, 2016). Notably, although there were some changes in diagnostic criteria for FC in Rome IV, the prevalence of FC in children was similar whether Rome IV or Rome III criteria were used (Tran and Sintusek, 2023). The pathophysiologic mechanisms of FC in children are generally recognized as complex and multifactorial, including disturbed gut microbiota, anorectal dysfunctions, stool withholding behavior, psychological issues, genetic predisposition, and diet (Levy et al., 2017). Stool withholding behavior is thought to be the main pathophysiological mechanism of FC in children. Incorrect bowel training, defecation in a standing position, and painful defecation with hard stools all tend to promote fecal retention in the rectum and lead to hard fecal clumps. The prevalence of fecal soiling, palpable fecal mass on abdominal examination, and withholding behavior in children with FC have been reported to be 33%-77%, 33%-68%, and 37%-91%, respectively (Tran and Sintusek, 2023). Therefore, oral laxatives and organized toilet training are the mainstays of treatment for FC in children (Tran and Sintusek, 2023). In addition, the role of the gut microbiome in the pathophysiology of FC is increasingly being emphasized. It has been shown that there are significant differences in the gut microbiota between healthy individuals and children with FC (Shin et al., 2019). The microbiome is potentially linked to intestinal motility. Alterations including delayed gastrointestinal transit, decreased frequency of fecal expulsion, and altered intestinal smooth muscle contractility have been reported in adolescent populations following antibiotic-induced dysbiosis (Ge et al., 2017). Similarly, mice treated with antibiotics exhibited lower intestinal and colonic transit times overall, as well as reduced levels of microbial-derived 5-hydroxytryptamine production (Caputi et al., 2017). Therefore, targeting the gut microbiota for the treatment of FC has become an area of great interest to researchers. Probiotic have been recognized as a promising therapy for the treatment of FC in children.

### 4.4 Promise of probiotics for FC in children

The gut microbiota plays a key role in the development and progression of constipation. As a result, therapies that turn to microecological interventions, especially probiotics, are receiving increasing attention (Pan et al., 2022). According to the included studies, the probiotic strains used for the intervention were primarily from the genera Lactobacillus and Bifidobacterium: *E. coli* Nissle 1917, *L. casei* Shirota*, L. rhamnosus* Lcr35, *B. lactis* DN-173 010, *Lactobacillus rhamnosus* GG, *Lactobacillus* Sporogenes, *B. breve, L.acidophilus, B. infantis, L. reuteri* DSM17938, *Lactobacillus reuteri, Lactobacillus rhamnosus*. *L. rhamnosus* Lcr35 was assessed by six included reviews, and the results suggested that patients who experienced probiotic treatment had significant improvements in both stool frequency and stool consistency. Similarly, *B. lactis* DN-173 010 was also assessed by six included reviews, and the results suggested that patients who experienced probiotic treatment had significant improvements in both stool frequency and stool consistency. Four included reviews evaluated *L. reuteri DSM17938*, and the results showed significant improvement in constipation symptoms in all patients treated with probiotics. As a lactic acid bacterium widely used in probiotic products, *Lactobacillus rhamnosus* GG was assessed by three studies and the results suggested that patients who experienced probiotic treatment had significant improvements in both stool frequency and stool consistency. Although both of these commonly used Lactobacillus and Bifidobacterium showed therapeutic potential for FC, the quality of evidence for their effect sizes was mostly rated as low, meaning that caution should be exercised when recommending these probiotics as treatments for FC in children. In addition, most of the studies in which probiotics were used to treat FC in children were heterogeneous in terms of probiotic strains, dosage, duration, and follow-up. Therefore, there is still an urgent need for further studies to determine the optimal probiotic strains, doses, and duration of use.

### 4.5 Strength and limitations

This overview critically evaluated and summarized the current evidence on probiotics for FC in children, and the results may facilitate clinical decision making for complementary therapies. However, some limitations should be acknowledged. First, in exploring the health issue of FC, the inclusion of studies has paid little attention to, if not outright ignored, factors such as diet, physical activity, and water intake, all of which may have influenced the true efficacy of probiotics. Second, there is a great lack of knowledge of microbial profiles in infants and the exact prescriptions of microbial strains that allow subjects to reestablish their metabolic homeostasis not only in population quantities but also in microbial interactions.

## 5 Conclusion

Probiotics may be beneficial in improving FC in children. Because of limitations and inconsistent conclusions, further rigorous, normative and comprehensive SRs/MAs are needed to provide robust evidence for definitive conclusions.

## Data availability statement

The original contributions presented in the study are included in the article/supplementary material. Further inquiries can be directed to the corresponding author.

## Author contributions

YZ: Conceptualization, Writing – original draft. AL: Conceptualization, Writing – original draft. JQ: Conceptualization, Data curation, Writing – original draft. HW: Conceptualization, Data curation, Writing – original draft. HZ: Conceptualization, Writing – original draft. XS: Conceptualization, Writing – original draft.
